# Supplementary material for: Diatom fucan polysaccharide precipitates carbon during algal blooms
Source: Nat Commun. 2021 Feb 19;12:1150. doi: 10.1038/s41467-021-21009-6 (PMC7896085; doi:10.1038/s41467-021-21009-6)
Supplement: Supplementary file 1 — Supplementary Information [file 41467_2021_21009_MOESM1_ESM.pdf]

Supplementary Information

Diatom fucan polysaccharide precipitates carbon during algal blooms

Silvia Vidal-Melgosa, Andreas Sichert, T. Ben Francis, Daniel Bartosik, Jutta Niggemann, Antje Wichels, William G.T. Willats, Bernhard M. Fuchs, Hanno Teeling, Dörte Becher, Thomas Schweder, Rudolf Amann, Jan-Hendrik Hehemann*

*Corresponding author. Email: jhhehemann@marum.de

This file includes:

Supplementary Discussion

Supplementary Figures 1 to 9

Supplementary Tables 1 to 3

Supplementary References

Supplementary Discussion

Spring bloom 2016 at station Kabeltonne

The sampling was conducted in the North Sea at station Kabeltonne (54˚11.3'N, 7˚54.0'E) approximately 60 km offshore from the northern German coastline near the island of Helgoland, Germany. The 2016 spring bloom at station Kabeltonne developed from the 08/03 until the 17/05, Julian days 68 and 138, respectively. During the field campaign chlorophyll a concentration fluctuated with values ranging between 2.1 and 11.8 mg m^{-3} (Supplementary Fig. 1b). The bloom was dominated by centric diatoms as well as by flagellates, while there were coccolithophorids, pennate diatoms, dinoflagellates and ciliates in lower abundance (most abundant taxa shown in Supplementary Fig. 2). Centric diatom cell numbers decreased notably on the 17/05 and on that same date there was a coccolithophorids peak. Regarding the phytoplankton that were identified at species or genus level (Supplementary Fig. 6a), *Chaetoceros* sp. were dominant during the bloom, which is also indicated by the remarkably similar abundance trend of total diatom centrales and *Chaetoceros* sp. (Supplementary Fig. 2 compared to Supplementary Fig. 6a). Diatoms use silicate to produce their frustule and we observed that silicate concentration decreased since the start of the diatom bloom with a vast diminution from day 29/03 (Julian day 89) (Supplementary Fig. 9). The concentration of phosphate (PO_4) markedly decreased from the start of the diatom bloom; while dissolved inorganic nitrogen (DIN) concentration also decreased during the bloom but not as severely as PO_4.

In order to perform a time course study of microalgae-produced polysaccharides we needed a water body predominantly stable over the sampling period. Station Kabeltonne has been previously described by Teeling and co-authors[1] as a station that "*provides rapid and easy access to coastal oceanic water that is far enough off the highly dynamic transient coast, but close enough to reside within the major coastal current that transports nutrients from continental European rivers*"[1]. In 2016, prior to the diatom bloom, on the 16/02 (Julian day 47) there was a clear presence of estuarine water masses (most likely from the German rivers Weser and Elbe) at station Kabeltonne, evidenced by a sharp decrease in salinity as well as a large increase in abundance of DIN and silicate (highest peaks) (Supplementary Fig. 9). During the sampling period - from 15/03 to 26/05 (Julian days 75 and 147) - the salinity remained constant (values between 31.5 and 33.7), indicating stability regarding the water body with few notable mentions: on days 21/03 and 24/03 (Julian days 81 and 84), 13 days after the start of the diatom

bloom, there was an influx from estuarine nutrient-rich water indicated by a decrease in salinity that coincided with peaks in DIN and silicate. This influx could have aided the diatom bloom as there was an increase in diatom cell numbers after it. There were two additional drops in salinity, one on the 27/04 (Julian day 118) that coincided with a DIN peak but no silicate increase and one on the 24/05 (Julian day 145) that did not result in an increase of DIN neither silicate. We observed two PO_4 peaks (14/04 and 18/04; Julian days 105 and 109) with no correlation with any of the physicochemical parameters and with no apparent effect on the phytoplankton composition.

Monosaccharide analysis comparison to previous HMWDOM studies

Our monosaccharide analysis data (Supplementary Fig. 3) show that the monomer composition in high molecular weight dissolved organic matter (HMWDOM) at the North Sea was relatively constant during the course of the bloom. The main monosaccharides were: glucuronic acid, xylose, mannose, glucose, galactose, glucosamine, arabinose and fucose. With the exception of rhamnose, our reported monosaccharide composition in tangential flow filtration-processed HMWDOM from surface seawater are comparable to previous monosaccharide composition analyses in ultrafiltered HMWDOM from surface seawater in geographically diverse sites including the Atlantic and Pacific oceans[2]. Aluwihare *et al*. data exclude acidic and amino sugars as they represented < 12% of the total[2]. For our results, the sum of acidic and amino sugars represented as well < 0.12 of the total when excluding glucosamine, which alone represented 0.14 ± 0.04.

Chemical characterisation by carbohydrate microarray analysis

We analysed several polysaccharide epitopes in parallel during a time series as they occurred in native particulate organic matter (POM) and HMWDOM samples from the ocean. To do this, we employed carbohydrate microarray technology whereby samples were printed at high spatial densities onto nitrocellulose membrane using a robot to achieve non-covalent immobilisation. These arrays were then probed with a collection of monoclonal antibody (mAb) and carbohydrate binding module (CBM) probes (listed in Supplementary Table 1). This is a semiquantitative method proven to be highly reproducible, robust and specific[3,4] (Supplementary Fig. 4a,b). The use of mAbs and CBMs for glycoprofiling is well established in plant science and this is reflected by the fact that the majority of probes were raised against, and bind to, polysaccharides prevalent in land plants. This limits our capacity to detect the entire

collection of algal polysaccharides from our samples. Nevertheless, some polysaccharides are common to both marine algae and land plants, either because of shared evolution of biosynthetic pathways or convergent evolution. For example, the β-1,3-glucan epitope is found in macro and microalgae (laminarin) as well as in land plants (callose). We demonstrated the novel application of microarray-based marine polysaccharide glycoprofiling and our data (Supplementary Fig. 5) reveal the presence of 27 discrete polysaccharide epitopes during the course of a microalgae bloom and we propose that although some glycans of bacterial or macroalgal origin theoretically may be present, the vast majority of polysaccharides detected were produced by microalgae. This microarray approach allows immobilisation of polysaccharides but not of monosaccharides and short oligosaccharides[4,5]. Therefore, as specified through the text, our data show polysaccharide epitopes present in HMWDOM and POM.

With this technique, the relative abundance of epitopes is determined by the signals reported by antibody binding and this relationship has previously been demonstrated[6]. We corroborated this by printing polysaccharide standards with serial dilutions, which after probing, resulted in mAb signals that correlated with epitope concentration (Supplementary Fig. 4a). Since different antibodies have different avidities (overall binding capacity), the signal intensities obtained with two different probes should not be used to infer differences in abundance of the corresponding epitopes. By contrast, the signal intensities from a single antibody can be used to determine the relative abundance of its recognised polysaccharide structure within a sample set. Our samples were normalised by weight (dry mass - see Methods), thus by examining the binding signal from each individual mAb we can monitor changes in epitope abundance within HMWDOM and within POM during the bloom (Supplementary Fig. 5). Note that the temporal dynamics but not the absolute number should be compared between HMWDOM and POM pools as their data sets required independent normalisation since their sampling was different. Also, in HMWDOM trends but not absolute numbers can be compared between water extracts (which had an extra 3-fold dilution) and EDTA plus NaOH extracts (see Methods).

Antibodies recognise unique epitopes of polysaccharides showing their abundance over time, however multiple factors may influence the detected abundances. We were following the presence of molecules in a dynamic environment with continuous microalgae-bacteria interactions, thus the decrease of a polysaccharide epitope may be due to extensive bacterial consumption but can also be due to modification by certain enzyme activities such as de-esterification. Also, although concentration changes are an important indicator of molecular

reactivity, physicochemical properties need to be considered to interpret polysaccharide abundance in HMWDOM and POM. For example, homogalacturonan (α-1,4-galacturonan), which was barely detected in HMWDOM (Fig. 1f), is a carboxylated and therefore cation chelating polysaccharide[7], rendering it insoluble in seawater with 10 mM calcium. Thus its lack in HMWDOM cannot be used to infer bacterial degradation. Instead low solubility is supported because it was only detected when extracted with the metal cation chelator EDTA (Supplementary Fig. 5). Likewise, the water insoluble cell wall polysaccharide cellulose was only detected in NaOH-solubilised extracts. NaOH is used to extract plant cell wall hemicelluloses[3,4,8] and hemicellulose-type epitopes, such as mixed-linkage glucan (β-1,3;1,4-glucan) and xyloglucan, were detected in HMWDOM and POM primarily when extracted with NaOH (Supplementary Fig. 5). In contrast, several epitopes were extracted with more than one solvent, possibly due to the effect of polysaccharide distinct length, branching pattern and cross-linking with other polymers. If certain polysaccharide types are cross-linked they will be held tighter to the polymer network than non-associated ones - such as one single polysaccharide type with different degree of branching influencing association with other polymers. In the case of HMWDOM, although most of the polysaccharides were found in the water extracts and that is why they had to be 3-fold diluted, there were glycans extracted with EDTA and NaOH. These polysaccharides were in the dissolved matter pool when harvested, however we extensively concentrated them during the sampling process and this could result in that a fraction of them started to form polymer networks. In addition, even though the HMWDOM pool contains dissolved matter, it also contains small marine colloids or particle precursors (< 0.2 μm) and our extensive concentration would enhance their aggregation. Another fact worth mentioning is the several cases where mAbs specific for one polysaccharide show different trends, for instance the arabinogalactan-specific mAbs JIM13 and MAC207, which reflect that each particular mAb recognises a unique particular epitope (Supplementary Fig. 5). Furthermore, the possibility of epitopes occurring on more than one type of polysaccharide, for example one particular epitope being present in two different algal polysaccharides, needs to be counted in.

Quantitative ELISA to investigate FCSP fold change in HMWDOM and POM

Our microarray results showed there was a higher abundance of the fucose-containing sulphated polysaccharide (FCSP) recognised by mAb BAM1 in HMWDOM at the beginning than at the end of the bloom, while in POM its presence was highest at the end (Supplementary Fig. 5). We investigated how much more FCSP there was at the beginning compared to the end, and

vice versa, in water extracts from both pools by using quantitative ELISA. Our reference standard was a fucan from macroalgae origin, fucoidan from the brown algae genus *Laminaria*, and thus we present data as ng macroalgal fucan equivalent per ml of extract (Supplementary Fig. 4c,d). Even though our standard fucan was from macroalgae, quantitative ELISA allowed us to provide the fold change of FCSP during the bloom, but absolute numbers may vary in dependence of the fucan standard used. For HMWDOM, comparing the concentration of macroalgal fucan equivalent in samples from the beginning and end, we found there was a 7-fold decrease during the bloom - ratio of average concentration of beginning-bloom to end-bloom samples. In POM size fraction between 10 and 3 μm, there was a 3-fold increase in concentration of macroalgal fucan equivalent - ratio of average concentration of end-bloom to beginning-bloom samples. When comparing the pools, there is 70 times more macroalgal fucan equivalent in HMWDOM extracts than in POM extracts - ratio of average concentration of six HMWDOM samples to six POM samples (for HMWDOM the 19.05 sample was excluded to compare 6 and 6 extracts). Clearly this last comparison does not reflect the real proportion of FCSP in both pools during the bloom as for this experiment all samples have been normalised by weight, while during the sampling campaign HMWDOM samples were heavily concentrated (100 L of 0.2 μm-filtered seawater concentrated to 0.5 L) compared to POM samples (see Methods).

Microarray epitopes compared to metagenomic results

Our carbohydrate microarray analysis revealed the presence of several polysaccharide structures in HMWDOM and POM during the course of the diatom bloom. Metagenomic analysis showed that the genomes of marine bacteria presented a large repertoire of genes coding for CAZymes that mediate polysaccharide degradation. In Supplementary Table 3 we summarise the epitopes detected by microarray analysis and list CAZyme families that we consider could potentially be able to degrade them (complete data sets in Supplementary Fig. 5 and 7a, respectively).

Supplementary Figures

a

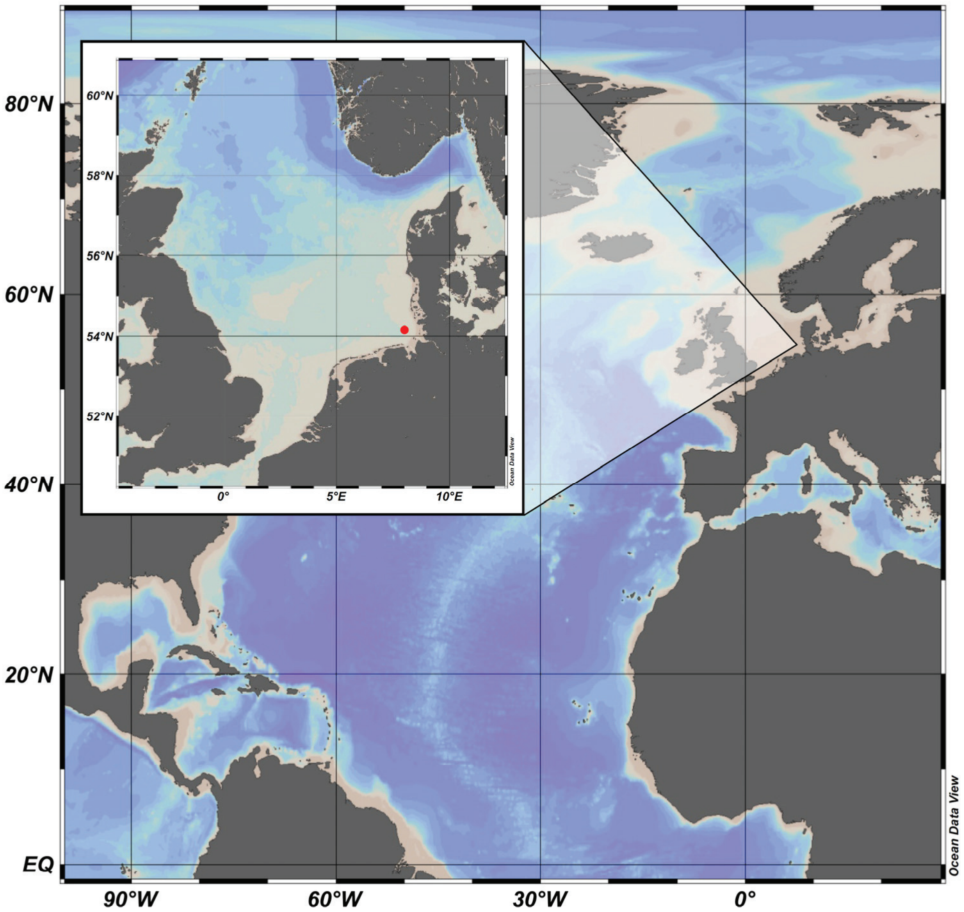

b

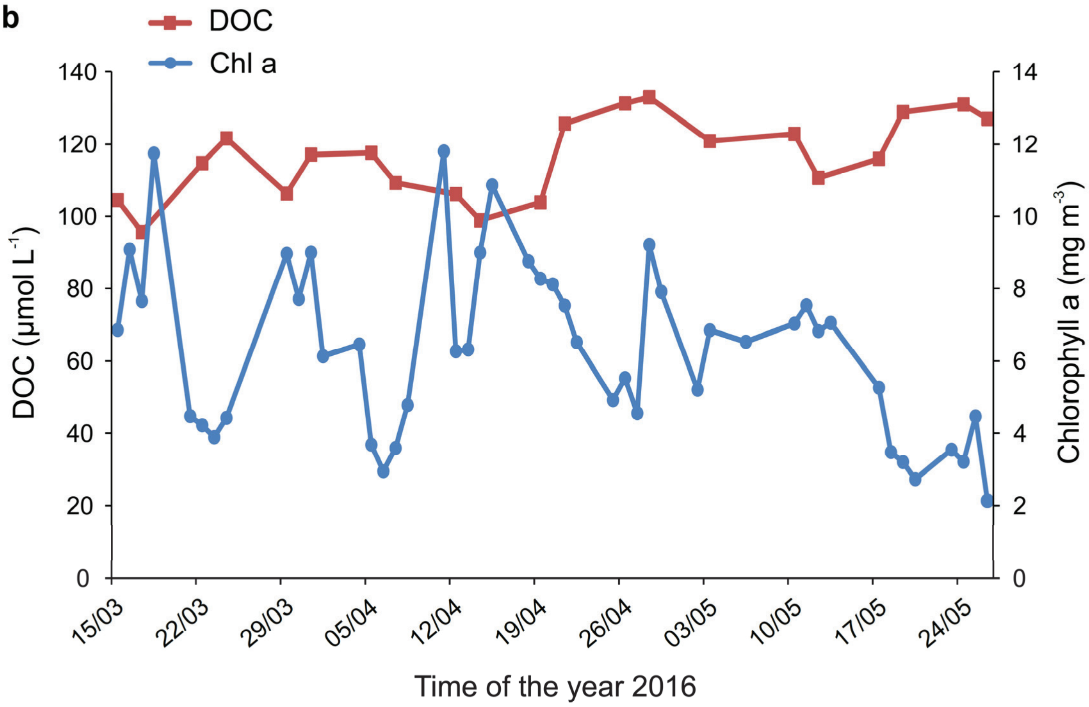

Supplementary Fig. 1. Sampling site, chlorophyll a and DOC values. a, Location of the sampling site (54˚11.3'N, 7˚54.0'E) in the German Bight of the North Sea indicated in the map with a red dot.

The map was created using Ocean Data View v5.2.1 (Schlitzer, Reiner, https://odv.awi.de, 2020). **b,** Values obtained from the analysis of dissolved organic carbon (DOC) in the DOM pool (< 0.2 µm) during the spring bloom in 2016. Chlorophyll a concentrations detected from mid-March until end of May are shown as well.

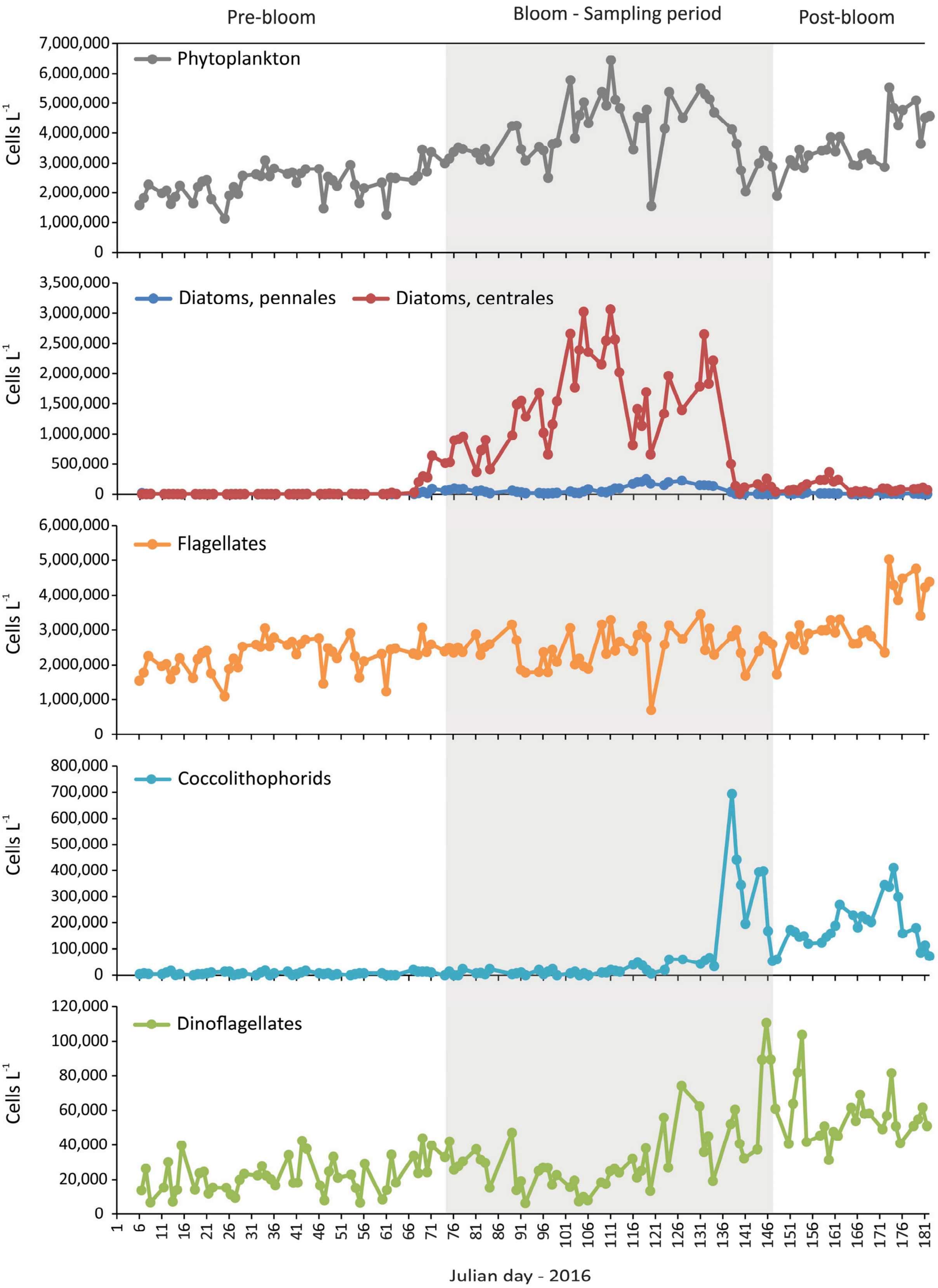

Supplementary Fig. 2. The most abundant phytoplankton taxa present during the spring bloom. Phytoplankton (on top) represents the sum of all taxa (including nano- and micro-phytoplankton)

detected from beginning of January to end of June 2016. Note that the diatom bloom initiated one week before the start of the sampling period.

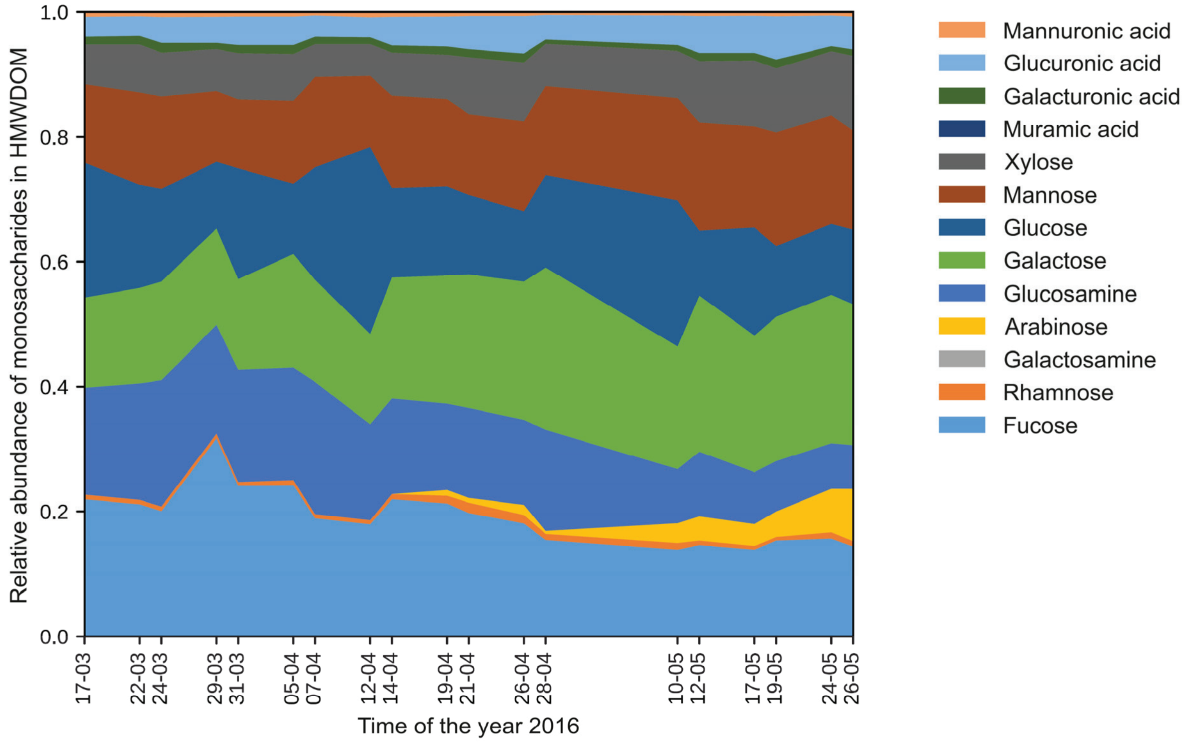

Supplementary Fig. 3. Compositional monosaccharide analysis of HMWDOM. Analysis of 19 HMWDOM samples from the bloom was performed by acid hydrolysis followed by quantification of monosaccharides using high performance anion exchange chromatography with pulsed amperometric detection (HPAEC-PAD). Data correspond to two technical replicates, n = 2. Concentration of monosaccharides is presented as mean relative abundance.

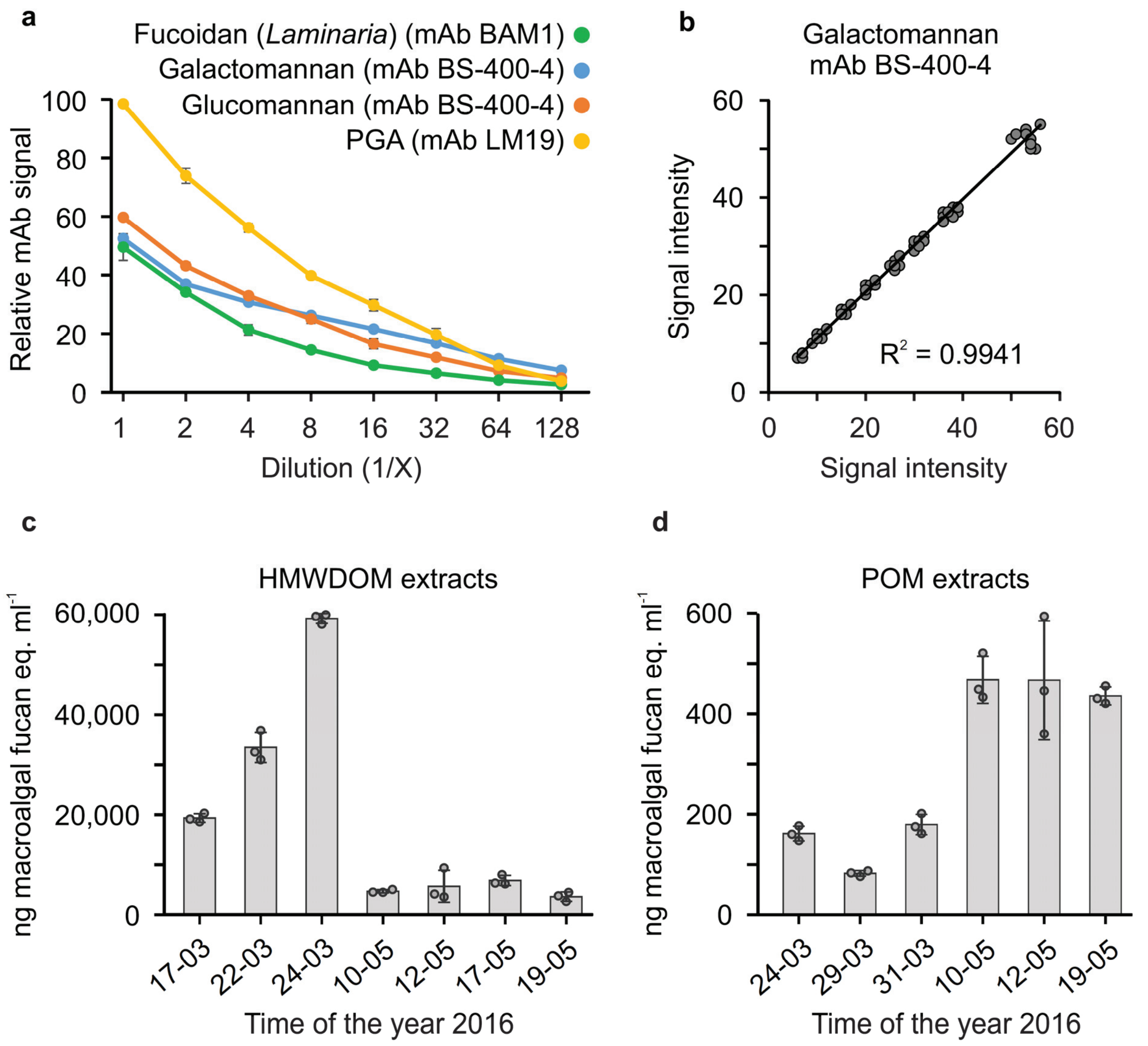

Supplementary Fig. 4. Substrate concentration effect on carbohydrate microarrays and FCSP fold change in HMWDOM and POM during the bloom. a, Plot presents the correlation between concentration of standard polysaccharides and monoclonal antibody (mAb) signal intensity, which progressively decreases with decreasing epitope concentration. Dots and error bars correspond to mean ± standard deviation of n = 12 replicates per each concentration. For the four polysaccharides first dilution was 1 mg ml^{-1}. PGA, polygalacturonic acid. **b,** Reproducibility of the microarrays tested by comparing the mAb signal intensities obtained from two individual microarrays probed with the same antibody. The four polysaccharide-mAb pairs from section (**a**) were tested and all comparisons (spot signals from each of the two microarrays probed with the same mAb plotted against each other) resulted in $R^2 > 0.99$ indicating a low level of variability. Representative graph shows galactomannan signals detected with mAb BS-400-4 in one array (*x* axis) and in the other array (*y* axis), n = 96 spotted galactomannan samples per array. **c-d,** Concentration of the FCSP recognised by mAb BAM1 in HMWDOM (**c**) and POM (**d**) water extracts was estimated by quantitative ELISA using fucoidan from the macroalgae genus *Laminaria* as standard. Amount of FCSP in the extracts is expressed as ng

macroalgal fucan equivalent per ml of extract. Sampling date is shown in *x* axis. POM extracts are from the size fraction between 10 and 3 µm. Data are mean ± standard deviation of n = 3 technical replicates.

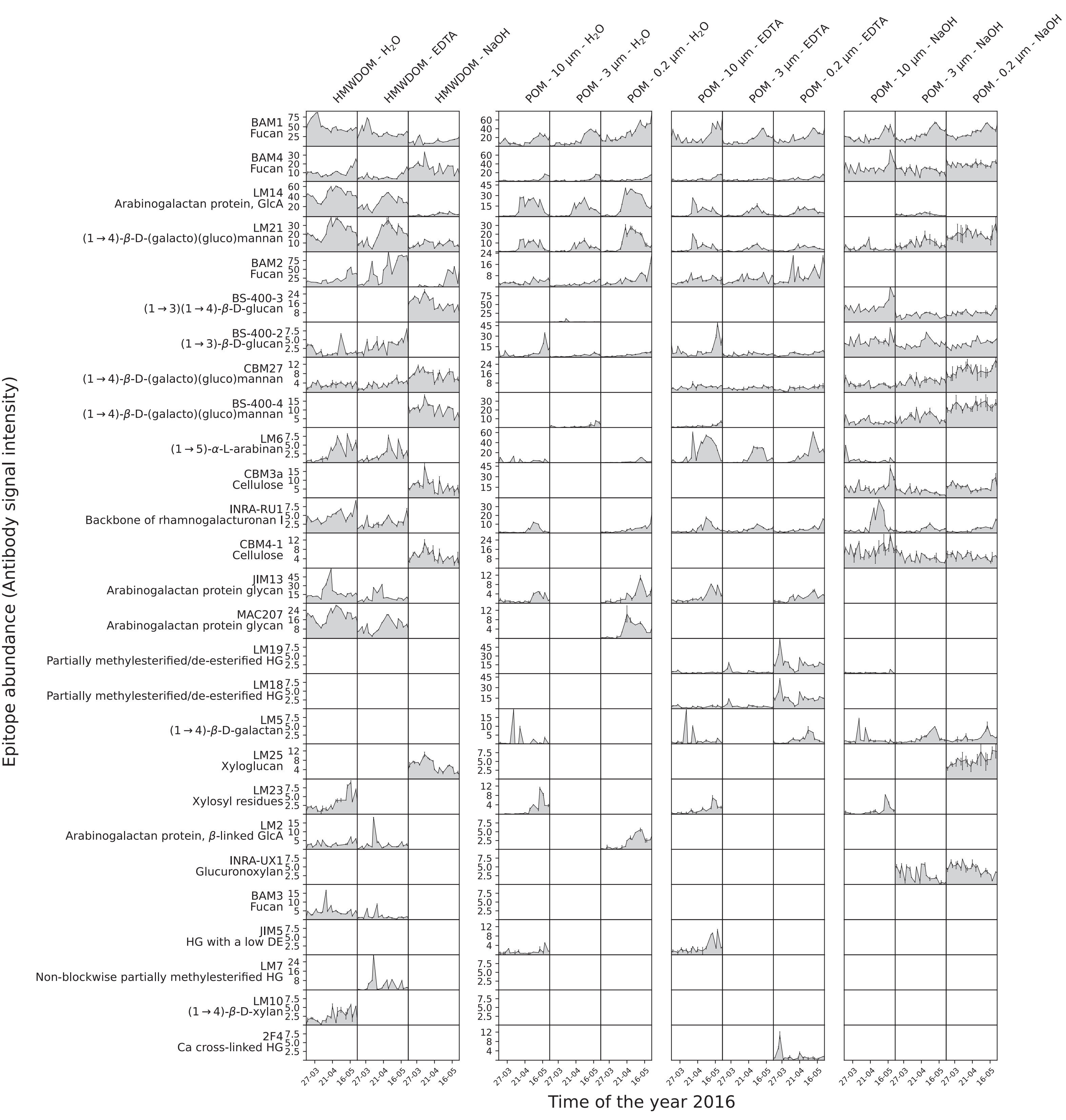

HMWDOM - H_2O
HMWDOM - EDTA
HMWDOM - NaOH
POM - 10 µm - H_2O
POM - 3 µm - H_2O
POM - 0.2 µm - H_2O
POM - 10 µm - EDTA
POM - 3 µm - EDTA
POM - 0.2 µm - EDTA
POM - 10 µm - NaOH
POM - 3 µm - NaOH
POM - 0.2 µm - NaOH
BAM1 Fucan
BAM4 Fucan
LM14 Arabinogalactan protein, GlcA
LM21 $(1\rightarrow4)$-β-D-(galacto)(gluco)mannan
BAM2 Fucan
BS-400-3 $(1\rightarrow3)(1\rightarrow4)$-$\beta$-D-glucan
BS-400-2 $(1\rightarrow3)$-β-D-glucan
CBM27 $(1\rightarrow4)$-β-D-(galacto)(gluco)mannan
BS-400-4 $(1\rightarrow4)$-β-D-(galacto)(gluco)mannan
LM6 $(1\rightarrow5)$-α-L-arabinan
CBM3a Cellulose
INRA-RU1 Backbone of rhamnogalacturonan I
CBM4-1 Cellulose
JIM13 Arabinogalactan protein glycan
MAC207 Arabinogalactan protein glycan
LM19 Partially methylesterified/de-esterified HG
LM18 Partially methylesterified/de-esterified HG
LM5 $(1\rightarrow4)$-β-D-galactan
LM25 Xyloglucan
LM23 Xylosyl residues
LM2 Arabinogalactan protein, β-linked GlcA
INRA-UX1 Glucuronoxylan
BAM3 Fucan
JIM5 HG with a low DE
LM7 Non-blockwise partially methylesterified HG
LM10 $(1\rightarrow4)$-β-D-xylan
2F4 Ca cross-linked HG
Epitope abundance (Antibody signal intensity)
27-03
21-04
16-05
Time of the year 2016

Supplementary Fig. 5. See figure in previous page.

Supplementary Fig. 5. Polysaccharide structures detected in HMWDOM and POM by carbohydrate microarray analysis. Carbohydrate microarray data showing the relative polysaccharide abundance (antibody signal intensity, *y* axis) detected in the different extracts (specific size fraction and extraction solvent depicted at the top) throughout the spring phytoplankton bloom (21 sampling dates, time in *x* axis). Binding for a total of 27 probes specific for particular polysaccharide epitopes (listed to the left) was detected. Spot signal intensities for each sample (each extract was represented by 4 spots in the array) against each probe were quantified and the highest mean signal value in the data set for HMWDOM and for POM was set to 100 and all other values were normalised accordingly. Data are mean values, n = 4 spots per extract. The temporal dynamics but not the absolute number should be compared between HMWDOM and POM pools as they required independent normalisation, since they required different sampling strategies. A cut-off of 5 arbitrary units was applied and figure shows all profiles where in at least one date an antibody positive signal (value ≥ 5) was detected. Size fractions correspond to: HMWDOM, between 0.2 µm and 1 kDa; 10 µm, over 10 µm; 3 µm, between 10 and 3 µm; 0.2 µm, between 3 and 0.2 µm. Probes are arranged from most to least abundant epitope. Details of probes used are provided in Supplementary Table 1. GlcA, glucuronic acid; HG, homogalacturonan; DE, degree of esterification. Error bars represent ± standard deviation.

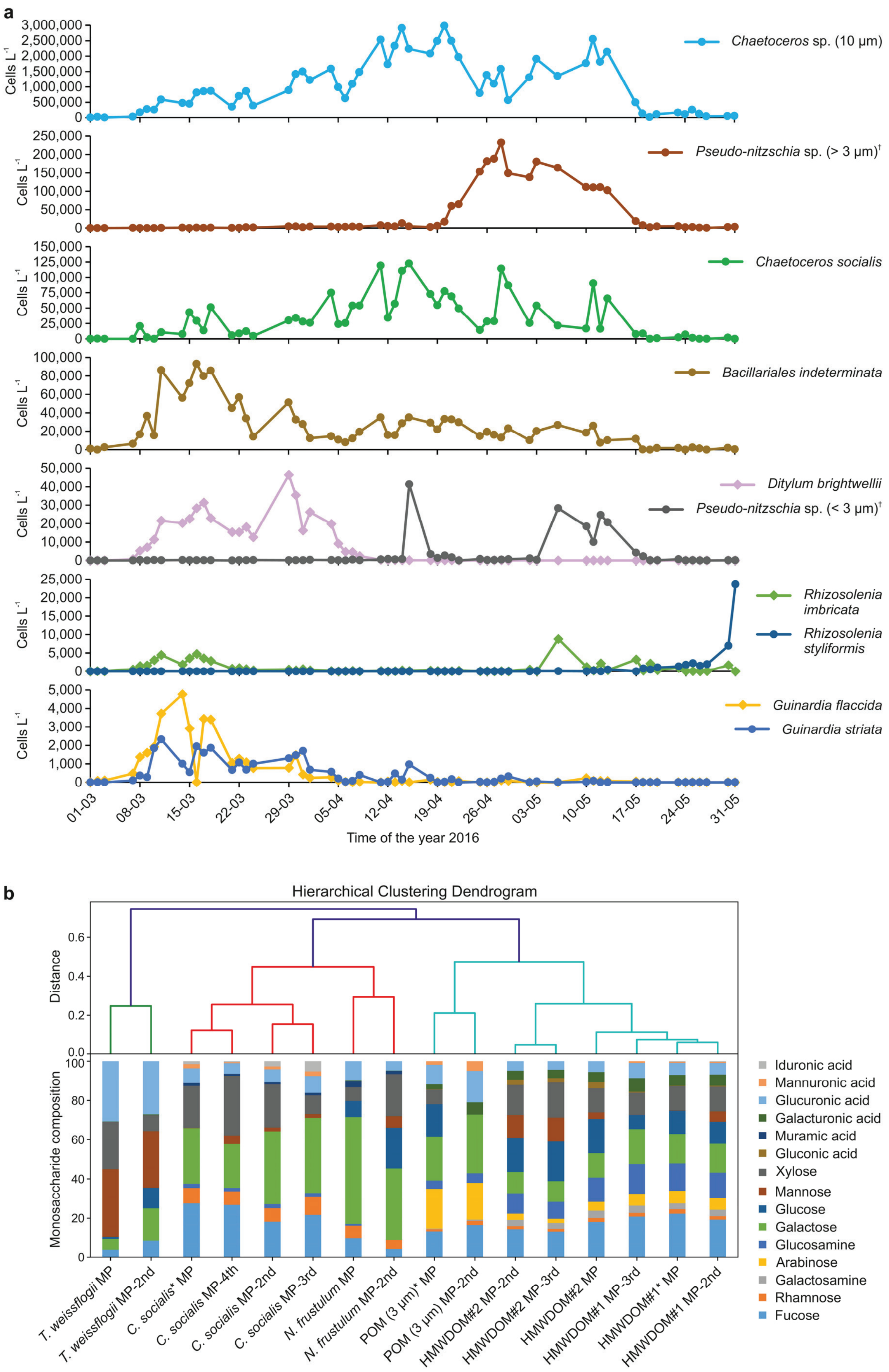
a
Cells L⁻¹
Chaetoceros sp. (10 µm)
Pseudo-nitzschia sp. (> 3 µm)†
Chaetoceros socialis
Bacillariales indeterminata
Ditylum brightwellii
Pseudo-nitzschia sp. (< 3 µm)†
Rhizosolenia imbricata
Rhizosolenia styliformis
Guinardia flaccida
Guinardia striata
01-03
08-03
15-03
22-03
29-03
05-04
12-04
19-04
26-04
03-05
10-05
17-05
24-05
31-05
Time of the year 2016
b
Hierarchical Clustering Dendrogram
Distance
Monosaccharide composition
T. weissflogii MP
T. weissflogii MP-2nd
C. socialis* MP
C. socialis MP-4th
C. socialis MP-2nd
C. socialis MP-3rd
N. frustulum MP
N. frustulum MP-2nd
POM (3 µm)* MP
POM (3 µm) MP-2nd
HMWDOM#2 MP-2nd
HMWDOM#2 MP-3rd
HMWDOM#2 MP
HMWDOM#1 MP-3rd
HMWDOM#1* MP
HMWDOM#1 MP-2nd
Iduronic acid
Mannuronic acid
Glucuronic acid
Galacturonic acid
Muramic acid
Gluconic acid
Xylose
Mannose
Glucose
Galactose
Glucosamine
Arabinose
Galactosamine
Rhamnose
Fucose

Supplementary Fig. 6. See figure in previous page.

Supplementary Fig. 6. Dominant phytoplankton species during the spring bloom and monosaccharide composition of purified FCSP. a, Cell counts of the ten most abundant phytoplankton that were identified at species or genus level in the sampling site from March to May 2016. †, *Pseudo-nitzschia* sp. > 3 µm refers to *Pseudo-nitzschia pungens* complex and *Pseudo-nitzschia* sp. < 3 µm refers to *Pseudo-nitzschia delicatissima* complex. **b**, Purified FCSP from different samples including: HMWDOM from the beginning of the bloom, POM from the end of the bloom, diatom monospecific laboratory cultures of *Thalassiosira weissflogii*, *Chaetoceros socialis* and *Nitzschia frustulum*. Polysaccharide extraction from the sample's biomass was performed. For each sample, the polysaccharides extracted with water were separated by anion exchange chromatography (AEC) and all AEC fractions were analysed by ELISA with the mAb BAM1 to determine the fractions containing purified FCSP (see Methods). AEC fractions with mAb BAM1 absorbance peak were acid hydrolysed and analysed by HPAEC-PAD. Monosaccharide composition of purified FCSP is presented as mean relative abundance. For each AEC fraction n = 2 independent acid hydrolysis. Samples are arranged according to a hierarchical clustering based on the Euclidean distance. MP, FCSP from the AEC fraction with BAM1 absorbance peak (main peak, highest BAM1 signal from ELISA); MP-2nd, FCSP from the AEC fraction that was part of the main peak (second highest BAM1 signal from the main peak); MP-3rd and MP-4th, when applicable, FCSP from the AEC fractions that were part of the main peak (third and fourth highest BAM1 signal from the main peak, respectively); #1 and #2, correspond to two separate experiments; *, FCSP from the fractions shown in Fig. 2g included here for comparison.

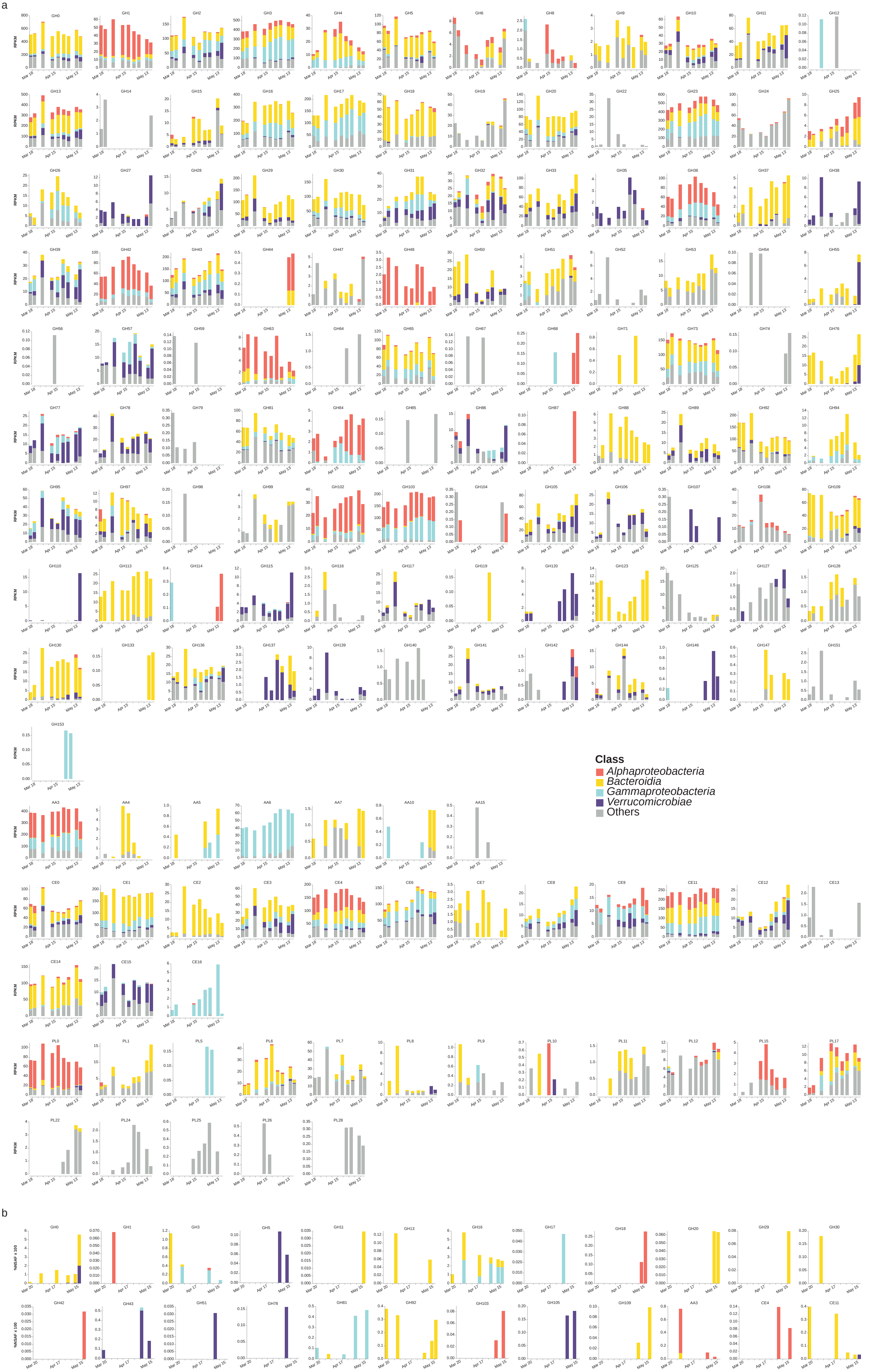
a
b
RPKM
%NSAF x 100
Mar 18
Apr 15
May 13
Mar 20
Apr 17
May 15
Class
Alphaproteobacteria
Bacteroidia
Gammaproteobacteria
Verrucomicrobiae
Others

Supplementary Fig. 7. See figure in previous page.

Supplementary Fig. 7. Complete CAZyme abundances and expression found by metagenomic and proteomic analyses during the phytoplankton bloom. Plots show abundances of genes coding for carbohydrate-active enzymes (CAZymes) with relevance for carbohydrate degradation in the genomes of marine bacteria (**a**) and their expression (**b**). Time of the year 2016 is shown in *x* axis. Family number 0 denotes CAZymes that have been assigned to an enzyme class but not yet assigned to a family. RPKM, reads per kilobase per million. Proteome data were analysed in a semiquantitative manner based on normalised spectral abundance factors (%NSAF). Both analyses include class-level taxonomic classifications, see Methods. GH, glycoside hydrolase; AA, auxiliary activity; CE, carbohydrate esterase; PL, polysaccharide lyase.

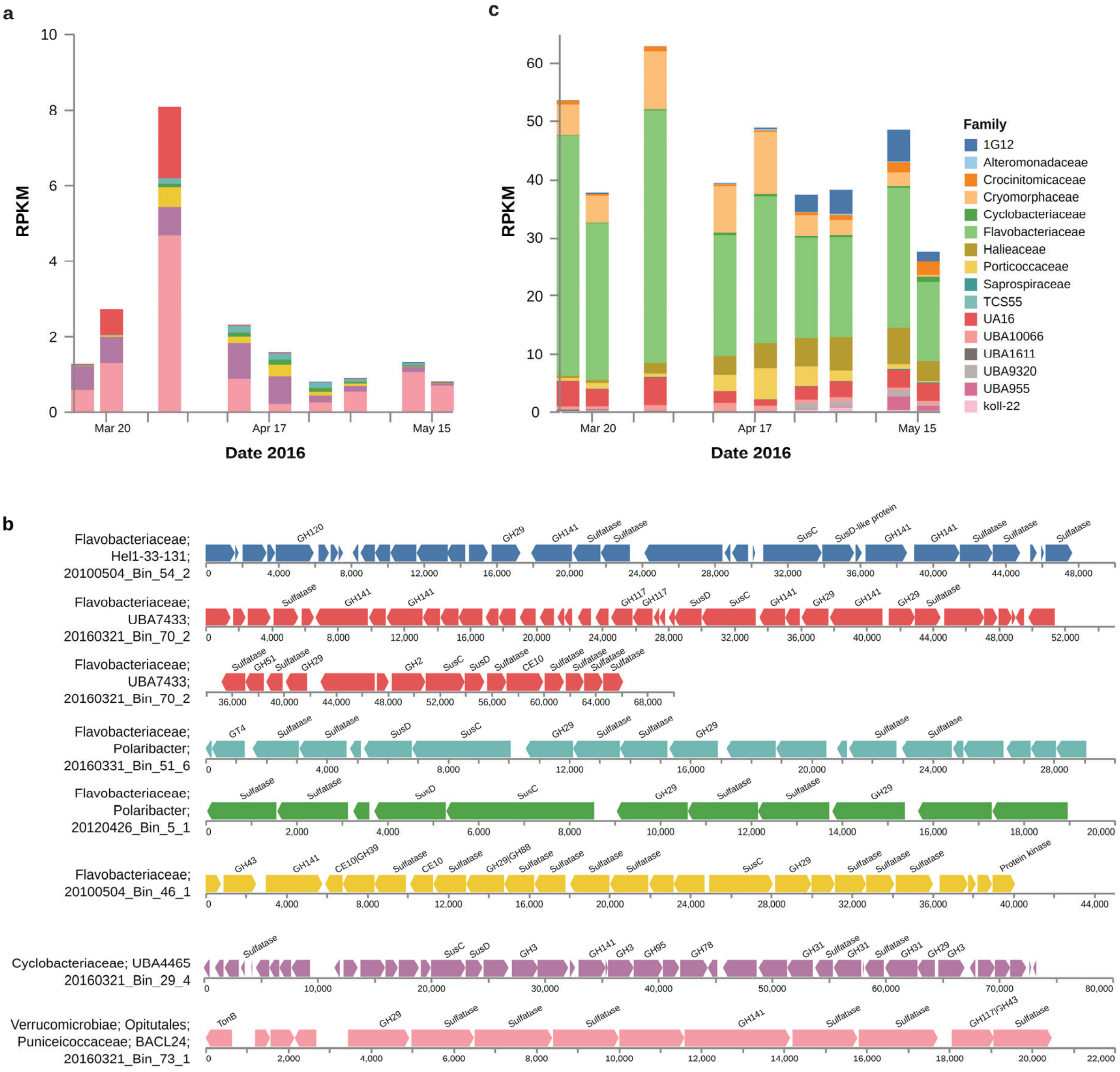

Supplementary Fig. 8. Presence of FCSP PULs during the phytoplankton bloom. a, Frequency of metagenome assembled genomes (MAGs) predicted to be able to consume FCSP, measured in reads per kilobase per million (RPKM). Colours are individual MAGs, corresponding to part (b). **b,** Gene organisation of putative FCSP polysaccharide utilisation loci (PULs). The two PULs belonging to *Flavobacteriaceae*-UBA7433-20160321_Bin_70_2 have the same RPKM as they derive from the same genome, and are thus represented once in the bar plot. **c,** RPKM of MAGs containing laminarin PULs, grouped at family level. All data are from MAGs derived from assembly of metagenomic data collected during spring blooms at Helgoland in the years 2010, 2011, 2012 and 2016.

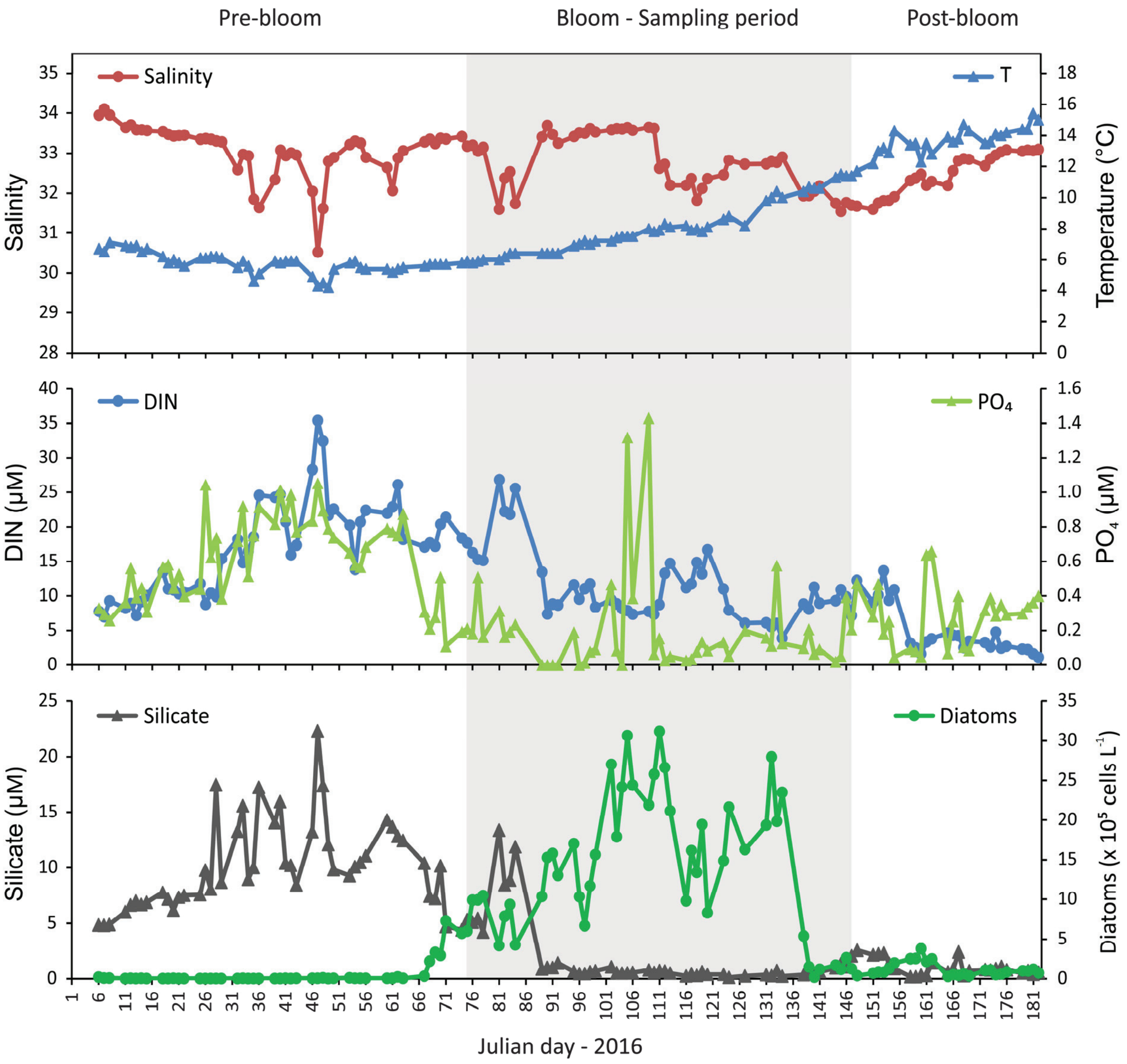

Supplementary Fig. 9. Major physicochemical parameters from beginning of January to end of June 2016. Measurements from subsurface seawater (54˚11.3'N, 7˚54.0'E). Diatoms abundance is shown together with silicate concentration to highlight the silicate decrease during the diatom bloom. DIN, dissolved inorganic nitrogen; PO_4, phosphate.

Supplementary Tables

Supplementary Table 1. Specificities of the probes used in this study

Probe	Recognised epitope structure	Binding on HMWDOM and/or POM	Reference
JIM5	Partially methyl-esterified/de-esterified HG*	YES	9
LM18	Partially methyl-esterified/de-esterified HG	YES	10
LM19	Partially methyl-esterified/de-esterified HG	YES	10
LM7	Non-blockwise partially methyl-esterified HG	YES	7
2F4	HG cross-linked through calcium ions	YES	11
INRA-RU1	Rhamnogalacturonan I backbone	YES	12
LM5	(1→4)-β-D-galactan	YES	13
LM6	(1→5)-α-L-arabinan	YES	14
LM21	(1→4)-β-D-(galacto)(gluco)mannan	YES	15
BS-400-4	(1→4)-β-D-(galacto)(gluco)mannan	YES	16
BS-400-2	(1→3)-β-D-glucan	YES	17
BS-400-3	(1→3)(1→4)-β-D-glucan	YES	18
LM25	Xyloglucan (XXXG motif, both galactosylated and non-galactosylated)	YES	5
LM10	(1→4)-β-D-xylan	YES	19
LM23	Xylosyl residues	YES	5
INRA-UX1	Glucuronoxylan	YES	20
MAC207	Arabinogalactan protein glycan	YES	21
LM2	β-linked GlcA in arabinogalactan protein	YES	22
LM14	GlcA in arabinogalactan protein	YES	5,23
JIM13	Arabinogalactan protein glycan	YES	24
BAM1	Un-sulphated epitope present in sulphated fucan	YES	25
BAM2	Sulphated epitope present in sulphated fucan	YES	25
BAM3	Possibly sulphated epitope present in sulphated fucan	YES	25
BAM4	Sulphated epitope present in sulphated fucan	YES	25
CBM3a	Cellulose	YES	26
CBM4-1	Cellulose	YES	27
CBM27	(1→4)-β-D-(galacto)(gluco)mannan	YES	28
JIM7	Partially methyl-esterified HG	NO	9
LM20	Partially methyl-esterified HG	NO	10
PAM1	Blockwise de-esterified HG	NO	29
LM8	Xylogalacturonan	NO	30
INRA-RU2	Rhamnogalacturonan I backbone	NO	12
LM12	Feruloylated polymers	NO	5
LM16	Galactosyl residue(s) on rhamnogalacturonan I	NO	31
LM13	Linearised (1→5)-α-L-arabinan	NO	31
LM22	(1→4)-β-D-(galacto)(gluco)mannan	NO	15
JIM6	Broad specificity for β-glucans (not to (1→3)-β-glucan)	NO	5
LM15	Xyloglucan (XXXG motif)	NO	32
LM24	Galactosylated xyloglucan	NO	5
CCRC-M1	Terminal fucosyl linked α-(1→2) to a galactosyl	NO	33
CCRC-M39	Fucosylated xyloglucan and rhamnogalacturonan I	NO	34
JIM4	Arabinogalactan protein glycan	NO	35
JIM8	Arabinogalactan protein glycan	NO	36
JIM14	Arabinogalactan protein glycan	NO	24
JIM16	Arabinogalactan protein glycan	NO	24
LM3	Extensin	NO	37
JIM20	Extensin	NO	38
INRA-COU1	*p*-coumaric acid and coumarate esters	NO	39
CBM30	(1→4)-β-glucopolymers	N.I.	40
LM11	(1→4)-β-D-xylan/arabinoxylan	N.I.	19
INRA-AX1	(1→4)-β-D-xylan/arabinoxylan	N.I.	41

*, preference in binding low methylated HG (for JIM5); HG, homogalacturonan; G, glucose; X, xylose; GlcA, glucuronic acid; N.I., not included in the results due to background noise signal. The list of probes includes monoclonal antibodies and carbohydrate binding modules (CBMs).

Supplementary Table 2. Presence of FCSP confirmed in different diatom species

Diatom species	Family	Order	Type
Chaetoceros socialis	*Chaetocerotaceae*	*Chaetocerotales*	Centric
Chaetoceros affinis	*Chaetocerotaceae*	*Chaetocerotales*	Centric
Chaetoceros debilis	*Chaetocerotaceae*	*Chaetocerotales*	Centric
Thalassiosira rotula	*Thalassiosiraceae*	*Thalassiosirales*	Centric
Thalassiosira weissflogii	*Thalassiosiraceae*	*Thalassiosirales*	Centric
Thalassiosira pseudonana	*Thalassiosiraceae*	*Thalassiosirales*	Centric
Nitzschia frustulum	*Bacillariaceae*	*Bacillariales*	Pennate
Phaeodactylum tricornutum	*Phaeodactylaceae*	*incertae sedis**	Pennate

*Bacillariophyta *ordo incertae sedis*. Diatom laboratory cultures were grown, biomass harvested and polysaccharides extracted as performed for *C. socialis* (described in Methods, sections Diatom laboratory cultures and FCSP separation by anion exchange chromatography). Presence of FCSP in each of the eight listed diatom species was confirmed with at least two of the following methods: carbohydrate microarray analysis, immunofluorescence microscopy, monosaccharide analysis, epitope detection chromatography and ELISA. mAb BAM1 and mAb BAM2 were used as detection tools. Presence of FCSP in diatom laboratory cultures was confirmed in the POM fraction as well as in the DOM fraction, where separation of the two fractions was done either by centrifugation (described in Methods, section Diatom laboratory cultures) or by filtration through 0.2 µm polycarbonate filters (47 mm diameter) at 200 mbar. Diatom taxonomic classification adopted from the database AlgaeBase, https://www.algaebase.org.

Supplementary Table 3. Polysaccharide epitopes compared to metagenomic results

Polysaccharide epitopes detected during the bloom	CAZyme families
Homogalacturonan ((1→4)-α-D-galacturonic acid) partially methyl-esterified and occasionally acetyl-esterified	PL1, PL9, PL10, GH28, GH105, CE8, CE12
Homogalacturonan ((1→4)-α-D-galacturonic acid) with no esterified groups	PL1, PL9, PL10, GH28, GH105
Rhamnogalacturonan I backbone (consists of the repeating disaccharide unit: [→4)-α-D-galacturonic acid-(1→2)-α-L-rhamnose-(1→])	PL1, PL9, PL10, PL26, GH28, GH78 GH105, CE8, CE12
(1→4)-β-D-galactan	GH35, GH42
(1→5)-α-L-arabinan	GH43
(1→4)-β-D-mannan	GH26, GH130
(1→4)-β-D-(galacto)(gluco)mannan: (1→4)-β-D-mannose backbone or (1→4)-β-D-mannose and D-glucose backbone. Mannan and glucomannan may be substituted with single (1→6)-α-D-galactose as side chain.	GH3, GH26, GH30, GH130
(1→3)-β-D-glucan	GH3, GH5, GH16, GH17, GH30
(1→3)(1→4)-β-D-glucan	GH3, GH5, GH16, GH30
Cellulose: (1→4)-β-D-glucan	GH3, GH5, GH16, GH30
Xyloglucan: (1→4)-β-D-glucan backbone branched with (1→6)-α-D-xylose residues that may be further substituted with (1→2)-β-D-galactose	GH3, GH5, GH10, GH11, GH16, GH30
(1→4)-β-D-xylan	GH10, GH11, GH43
Xylosyl residues	Not known
(1→4)-β-D-xylan backbone with some of the xylose units substituted with single α-D-glucuronic acid	GH10, GH11, GH43
Arabinogalactan protein glycan (most of them contain both arabinan and (1→3)-β-D-galactan and (1→6)-β-D-galactan chains substituted with terminal α-L-arabinose)	GH35, GH42, GH43
Arabinogalactan protein glycan with side chains containing D-glucuronic acid	GH35, GH42, GH43
Fucan: (1→3) or (1→3)(1→4)-α-L-fucose backbone extensively sulphated that may have side chains containing fucose, xylose and other monomers	GH29, GH95, GH107, GH141

Polysaccharide epitopes detected during the bloom by microarray analysis and CAZyme families in the genomes of bacterioplankton (metagenomics) that could putatively target them.

Supplementary References

1. Teeling, H. *et al.* Substrate-controlled succession of marine bacterioplankton populations induced by a phytoplankton bloom. *Science* **336**, 608–611 (2012).

2. Aluwihare, L. I., Repeta, D. J. & Chen, R. F. A major biopolymeric component to dissolved organic carbon in surface sea water. *Nature* **387**, 166–169 (1997).

3. Moller, I. *et al.* High-throughput mapping of cell-wall polymers within and between plants using novel microarrays. *Plant J.* **50**, 1118–1128 (2007).

4. Vidal-Melgosa, S. *et al.* A new versatile microarray-based method for high throughput screening of carbohydrate-active enzymes. *J. Biol. Chem.* **290**, 9020–9036 (2015).

5. Pedersen, H. L. *et al.* Versatile high resolution oligosaccharide microarrays for plant glycobiology and cell wall research. *J. Biol. Chem.* **287**, 39429–39438 (2012).

6. Øbro, J. *et al.* High-throughput microarray analysis of pectic polymers by enzymatic epitope deletion. *Carbohydr. Polym.* **70**, 77–81 (2007).

7. Willats, W. G. T. *et al.* Modulation of the degree and pattern of methyl-esterification of pectic homogalacturonan in plant cell walls: implications for pectin methyl esterase action, matrix properties, and cell adhesion. *J. Biol. Chem.* **276**, 19404–19413 (2001).

8. Kračun, S. K. *et al.* Carbohydrate microarray technology applied to high-throughput mapping of plant cell wall glycans using comprehensive microarray polymer profiling (CoMPP). in *High-Throughput Glycomics and Glycoproteomics* vol. 1503 147–165 (Humana Press, New York, 2017).

9. Clausen, M. H., Willats, W. G. T. & Knox, J. P. Synthetic methyl hexagalacturonate hapten inhibitors of anti-homogalacturonan monoclonal antibodies LM7, JIM5 and JIM7. *Carbohydr. Res.* **338**, 1797–1800 (2003).

10. Verhertbruggen, Y., Marcus, S. E., Haeger, A., Ordaz-Ortiz, J. J. & Knox, J. P. An extended set of monoclonal antibodies to pectic homogalacturonan. *Carbohydr. Res.* **344**, 1858–1862 (2009).

11. Liners, F., Letesson, J.-J., Didembourg, C. & Van Cutsem, P. Monoclonal antibodies against pectin: recognition of a conformation induced by calcium. *Plant Physiol.* **91**, 1419–1424 (1989).

12. Ralet, M.-C., Tranquet, O., Poulain, D., Moïse, A. & Guillon, F. Monoclonal antibodies to rhamnogalacturonan I backbone. *Planta* **231**, 1373–1383 (2010).

13. Jones, L., Seymour, G. B. & Knox, J. P. Localization of pectic galactan in tomato cell walls using a monoclonal antibody specific to (1→4)-β-D-galactan. *Plant Physiol.* **113**, 1405–1412 (1997).

14. Willats, W. G. T., Marcus, S. E. & Knox, J. P. Generation of a monoclonal antibody specific to (1→5)-alpha-L-arabinan. *Carbohydr. Res.* **308**, 149–152 (1998).

15. Marcus, S. E. *et al.* Restricted access of proteins to mannan polysaccharides in intact plant cell walls. *Plant J.* **64**, 191–203 (2010).

16. Pettolino, F. A. *et al.* A (1→4)-β-mannan-specific monoclonal antibody and its use in the immunocytochemical location of galactomannans. *Planta* **214**, 235–242 (2001).

17. Meikle, P. J., Bonig, I., Hoogenraad, N. J., Clarke, a E. & Stone, B. a. The location of (1→3)-β-glucans in the walls of pollen tubes of Nicotiana alata using a (1→3)-β-glucan-specific monoclonal antibody. *Planta* **185**, 1–8 (1991).

18. Meikle, P. J., Hoogenraad, N. J., Bonig, I., Clarke, A. E. & Stone, B. A. A (1→3,1→4)-β-glucan-specific monoclonal antibody and its use in the quantitation and immunocyto-chemical location of (1→3,1→4)-β-glucans. *Plant J.* **5**, 1–9 (1994).

19. McCartney, L., Marcus, S. E. & Knox, J. P. Monoclonal antibodies to plant cell wall xylans and arabinoxylans. *J. Histochem. Cytochem.* **53**, 543–546 (2005).

20. Koutaniemi, S. *et al.* Substituent-specific antibody against glucuronoxylan reveals close association of glucuronic acid and acetyl substituents and distinct labeling patterns in tree species. *Planta* **236**, 739–751 (2012).

21. Pennell, R. I., Knox, J. P., Scofield, G. N., Selvendran, R. R. & Roberts, K. A family of abundant plasma membrane-associated glycoproteins related to the arabinogalactan proteins is unique to flowering plants. *J. Cell Biol.* **108**, 1967–1977 (1989).

22. Yates, E. A. *et al.* Characterization of carbohydrate structural features recognized by anti-arabinogalactan-protein monoclonal antibodies. *Glycobiology* **6**, 131–139 (1996).

23. Moller, I. *et al.* High-throughput screening of monoclonal antibodies against plant cell wall glycans by hierarchical clustering of their carbohydrate microarray binding

profiles. *Glycoconj. J.* **25**, 37–48 (2008).

24. Knox, J. P., Linstead, P. J., Peart, J., Cooper, C. & Roberts, K. Developmentally regulated epitopes of cell surface arabinogalactan proteins and their relation to root tissue pattern formation. *Plant J.* **1**, 317–326 (1991).

25. Torode, T. A. *et al.* Monoclonal antibodies directed to fucoidan preparations from brown algae. *PLoS One* **10**, e0118366 (2015).

26. Blake, A. W. *et al.* Understanding the biological rationale for the diversity of cellulose-directed carbohydrate-binding modules in prokaryotic enzymes. *J. Biol. Chem.* **281**, 29321–29329 (2006).

27. Tomme, P., Creagh, A. L., Kilburn, D. G. & Haynes, C. A. Interaction of polysaccharides with the N-terminal cellulose-binding domain of Cellulomonas fimi CenC. 1. Binding specificity and calorimetric analysis. *Biochemistry* **35**, 13885–13894 (1996).

28. Boraston, A. B., Revett, T. J., Boraston, C. M., Nurizzo, D. & Davies, G. J. Structural and thermodynamic dissection of specific mannan recognition by a carbohydrate binding module, TmCBM27. *Structure* **11**, 665–675 (2003).

29. Willats, W. G. T., Gilmartin, P. M., Mikkelsen, J. D. & Knox, J. P. Cell wall antibodies without immunization: generation and use of de-esterified homogalacturonan block-specific antibodies from a naive phage display library. *Plant J.* **18**, 57–65 (1999).

30. Willats, W. G. T. *et al.* A xylogalacturonan epitope is specifically associated with plant cell detachment. *Planta* **218**, 673–681 (2004).

31. Verhertbruggen, Y. *et al.* Developmental complexity of arabinan polysaccharides and their processing in plant cell walls. *Plant J.* **59**, 413–425 (2009).

32. Marcus, S. E. *et al.* Pectic homogalacturonan masks abundant sets of xyloglucan epitopes in plant cell walls. *BMC Plant Biol.* **8**, (2008).

33. Puhlmann, J. *et al.* Generation of monoclonal antibodies against plant cell-wall polysaccharides. *Plant Physiol.* **104**, 699–710 (1994).

34. Pattathil, S. *et al.* A comprehensive toolkit of plant cell wall glycan-directed monoclonal antibodies. *Plant Physiol.* **153**, 514–525 (2010).

35. Knox, J. P., Day, S. & Roberts, K. A set of cell surface glycoproteins forms an early marker of cell position, but not cell type, in the root apical meristem of Daucus carota L. *Development* **106**, 47–56 (1989).

36. Pennell, R. I. *et al.* Developmental regulation of a plasma membrane arabinogalactan protein epitope in oilseed rape flowers. *Plant Cell* **3**, 1317–1326 (1991).

37. Smallwood, M., Martin, H. & Knox, J. P. An epitope of rice threonine- and hydroxyproline-rich glycoprotein is common to cell wall and hydrophobic plasma-membrane glycoproteins. *Planta* **196**, 510–522 (1995).

38. Smallwood, M. *et al.* Localization of cell wall proteins in relation to the developmental anatomy of the carrot root apex. *Plant J.* **5**, 237–246 (1994).

39. Tranquet, O., Saulnier, L., Utille, J.-P., Ralph, J. & Guillon, F. Monoclonal antibodies to p-coumarate. *Phytochemistry* **70**, 1366–1373 (2009).

40. Najmudin, S. *et al.* Xyloglucan is recognized by carbohydrate-binding modules that interact with beta-glucan chains. *J. Biol. Chem.* **281**, 8815–8828 (2006).

41. Guillon, F. *et al.* Generation of polyclonal and monoclonal antibodies against arabinoxylans and their use for immunocytochemical location of arabinoxylans in cell walls of endosperm of wheat. *J. Cereal Sci.* **40**, 167–182 (2004).
