# Supplementary material for: Diatom fucan polysaccharide precipitates carbon during algal blooms
Source: Nat Commun. 2021 Feb 19;12:1150. doi: 10.1038/s41467-021-21009-6 (PMC7896085; doi:10.1038/s41467-021-21009-6)
Supplement: Supplementary file 4 — Reporting Summary [file 41467_2021_21009_MOESM4_ESM.pdf]

nature research

Corresponding author(s): Jan-Hendrik Hehemann

Last updated by author(s): Dec 20, 2020

Reporting Summary

Nature Research wishes to improve the reproducibility of the work that we publish. This form provides structure for consistency and transparency in reporting. For further information on Nature Research policies, see our Editorial Policies and the Editorial Policy Checklist.

Statistics

For all statistical analyses, confirm that the following items are present in the figure legend, table legend, main text, or Methods section.

n/a	Confirmed	
☐	☒	The exact sample size (*n*) for each experimental group/condition, given as a discrete number and unit of measurement
☐	☒	A statement on whether measurements were taken from distinct samples or whether the same sample was measured repeatedly
☐	☒	The statistical test(s) used AND whether they are one- or two-sided<br>*Only common tests should be described solely by name; describe more complex techniques in the Methods section.*
☒	☐	A description of all covariates tested
☒	☐	A description of any assumptions or corrections, such as tests of normality and adjustment for multiple comparisons
☐	☒	A full description of the statistical parameters including central tendency (e.g. means) or other basic estimates (e.g. regression coefficient) AND variation (e.g. standard deviation) or associated estimates of uncertainty (e.g. confidence intervals)
☐	☒	For null hypothesis testing, the test statistic (e.g. *F*, *t*, *r*) with confidence intervals, effect sizes, degrees of freedom and *P* value noted<br>*Give P values as exact values whenever suitable.*
☒	☐	For Bayesian analysis, information on the choice of priors and Markov chain Monte Carlo settings
☒	☐	For hierarchical and complex designs, identification of the appropriate level for tests and full reporting of outcomes
☒	☐	Estimates of effect sizes (e.g. Cohen's *d*, Pearson's *r*), indicating how they were calculated

Our web collection on statistics for biologists contains articles on many of the points above.

Software and code

Policy information about availability of computer code

Data collection	Chromeleon v7.2, MagIC Net v3.2, Array-Pro Analyzer v6.3, MARS v3.01.R2, Zeiss AxioVision v4.8, ZEN black v2.1 (with service pack 3), ZEN blue v2.3.
Data analysis	SciPy v1.4.1, Python v3.7, BBDuk v35.14, metaSPAdes v3.10.0, Prodigal v2.6.3, HMMer v3.2.1, dbCAN v6, Diamond v0.9, BBMap v35.14, GTDB-Tk v0.3.1, GTDB v89, Mascot v2.6.0, Scaffold v4.8.6, X! Tandem vCYCLONE (2010.12.01.1), BLAST v2.6 and v2.9.0, Prokka v1.2.

For manuscripts utilizing custom algorithms or software that are central to the research but not yet described in published literature, software must be made available to editors and reviewers. We strongly encourage code deposition in a community repository (e.g. GitHub). See the Nature Research guidelines for submitting code & software for further information.

Data

Policy information about availability of data

All manuscripts must include a data availability statement. This statement should provide the following information, where applicable:

- Accession codes, unique identifiers, or web links for publicly available datasets
- A list of figures that have associated raw data
- A description of any restrictions on data availability

Glycan analyses data are available in PANGAEA, the carbohydrate microarray data are available at https://doi.pangaea.de/10.1594/PANGAEA.924287 and the monosaccharide composition data are available at https://doi.pangaea.de/10.1594/PANGAEA.924264. Metagenome assemblies and metagenome assembled genomes are available in the European Nucleotide Archive project PRJEB28156 [https://www.ebi.ac.uk/ena/browser/view/PRJEB28156]. Proteome mass spectral data are available in the ProteomeXchange Consortium via the PRIDE partner repository with the identifier PXD019294 [http://www.ebi.ac.uk/pride/archive/projects/PXD019294]. All other data are available within the article and supplementary information files or from the corresponding author on reasonable request.

The publicly available databases dbCAN v6 [http://bcb.unl.edu/dbCAN2/download/Databases/dbCAN-HMMdb-V6.txt] and CAZy database v07312018 [http://bcb.unl.edu/dbCAN2/download/CAZyDB.07312018.fa] were used for metagenome and metaproteome analyses.

Field-specific reporting

Please select the one below that is the best fit for your research. If you are not sure, read the appropriate sections before making your selection.

☒ Life sciences ☐ Behavioural & social sciences ☐ Ecological, evolutionary & environmental sciences

For a reference copy of the document with all sections, see nature.com/documents/nr-reporting-summary-flat.pdf

Life sciences study design

All studies must disclose on these points even when the disclosure is negative.

Sample size	No sample-size calculations were performed. The sampling period was determined based on data from Helgoland Roads LTER that showed the time when spring diatom blooms occurred in Helgoland in previous years.<br>For the omics analyses, we chose 9 dates for metagenomes and 6 dates for metaproteomes with samples across the whole sampling period to capture as faithfully as possible the dynamics of the bacterial community. Previous work has indicated that this sampling frequency is sufficient to capture the overall trends (e.g. Teeling et al. 2012, Teeling et al. 2016, Krüger et al. 2019).<br>For the polysaccharide analysis there were no previous studies regarding polysaccharide profiling. Therefore, in order to ensure an adequate sample size to assess glycan dynamics, we decided on a high sampling frequency of two times per week resulting in a total of 21 dates. Our results demonstrate that the sample size was sufficient.
Data exclusions	No data were excluded from the analyses.
Replication	We have verified the finding of FCSP accumulation in POM over the course of the bloom with three different methods: carbohydrate microarray analysis, immunofluorescence microscopy and quantitative ELISA. All attempts at replication were successful. Immunofluorescence microscopy experiments were performed four times including replicates and filters from six different dates (dates stated in Immunolabelling section in Methods), which resulted in comparable results between experiments. FCSP separation by anion exchange chromatography plus ELISA analyses of extracts from POM and HMWDOM were performed two times and four times respectively (for both samples from beginning and end of the bloom) and for extracts from diatom cultures analyses were repeated four times, all with similar results. Our experiments included biological or technical replicates (described in figure legends) to ensure the accuracy of data.
Randomization	No randomization was used in this study, since it was not applicable for our time-series. Our samples were always harvested from the same specific site and their allocation was by date.
Blinding	Investigators were not blinded. During sampling it was not possible because samples were not divided into groups but were divided by sampling date. No potential experimental biases were anticipated for our set of experiments as samples were not divided into treatment groups.

Reporting for specific materials, systems and methods

We require information from authors about some types of materials, experimental systems and methods used in many studies. Here, indicate whether each material, system or method listed is relevant to your study. If you are not sure if a list item applies to your research, read the appropriate section before selecting a response.

Materials & experimental systems

n/a	Involved in the study	
☐	☒	Antibodies
☒	☐	Eukaryotic cell lines
☒	☐	Palaeontology and archaeology
☒	☐	Animals and other organisms
☒	☐	Human research participants
☒	☐	Clinical data
☒	☐	Dual use research of concern

Methods

n/a	Involved in the study	
☒	☐	ChIP-seq
☒	☐	Flow cytometry
☒	☐	MRI-based neuroimaging

Antibodies

Antibodies used	Antibodies used in this study listed as: antibody name, source and antibody type, supplier, catalog number (when applicable).<br><br>- JIM5, Rat monoclonal, PlantProbes (Leeds, UK), Cat. No. JIM5<br>- LM18, Rat monoclonal, PlantProbes (Leeds, UK), Cat. No. LM18<br>- LM19, Rat monoclonal, PlantProbes (Leeds, UK), Cat. No. LM19<br>- LM7, Rat monoclonal, PlantProbes (Leeds, UK), Cat. No. LM7<br>- 2F4, Mouse monoclonal, PlantProbes (Leeds, UK), Cat. No. 2F4

- INRA-RU1, Mouse monoclonal, Institut National de la Recherche Agronomique (Nantes, France).
- LM5, Rat monoclonal, PlantProbes (Leeds, UK), Cat. No. LM5
- LM6, Rat monoclonal, PlantProbes (Leeds, UK), Cat. No. LM6
- LM21, Rat monoclonal, PlantProbes (Leeds, UK), Cat. No. LM21
- BS-400-4, Mouse monoclonal, BioSupplies (Bundoora, Australia), Cat. No. 400-4
- BS-400-2, Mouse monoclonal, BioSupplies (Bundoora, Australia), Cat. No. 400-2
- BS-400-3, Mouse monoclonal, BioSupplies (Bundoora, Australia), Cat. No. 400-3
- LM25, Rat monoclonal, PlantProbes (Leeds, UK), Cat. No. LM25
- LM10, Rat monoclonal, PlantProbes (Leeds, UK), Cat. No. LM10
- LM23, Rat monoclonal, PlantProbes (Leeds, UK), Cat. No. LM23
- INRA-UX1, Mouse monoclonal, Institut National de la Recherche Agronomique (Nantes, France).
- MAC207, Rat monoclonal, PlantProbes (Leeds, UK), Cat. No. MAC207
- LM2, Rat monoclonal, PlantProbes (Leeds, UK), Cat. No. LM2
- LM14, Rat monoclonal, PlantProbes (Leeds, UK), Cat. No. LM14
- JIM13, Rat monoclonal, PlantProbes (Leeds, UK), Cat. No. JIM13
- BAM1, Rat monoclonal, PlantProbes (Leeds, UK), Cat. No. BAM1
- BAM2, Rat monoclonal, PlantProbes (Leeds, UK), Cat. No. BAM2
- BAM3, Rat monoclonal, PlantProbes (Leeds, UK), Cat. No. BAM3
- BAM4, Rat monoclonal, PlantProbes (Leeds, UK), Cat. No. BAM4
- JIM7, Rat monoclonal, PlantProbes (Leeds, UK), Cat. No. JIM7
- LM20, Rat monoclonal, PlantProbes (Leeds, UK), Cat. No. LM20
- PAM1, Phage display-derived monoclonal, PlantProbes (Leeds, UK), Cat. No. PAM1
- LM8, Rat monoclonal, PlantProbes (Leeds, UK), Cat. No. LM8
- INRA-RU2, Mouse monoclonal, Institut National de la Recherche Agronomique (Nantes, France).
- LM12, Rat monoclonal, PlantProbes (Leeds, UK), Cat. No. LM12
- LM16, Rat monoclonal, PlantProbes (Leeds, UK), Cat. No. LM16
- LM13, Rat monoclonal, PlantProbes (Leeds, UK), Cat. No. LM13
- LM22, Rat monoclonal, PlantProbes (Leeds, UK), Cat. No. LM22
- JIM6, Rat monoclonal, PlantProbes (Leeds, UK), Cat. No. JIM6
- LM15, Rat monoclonal, PlantProbes (Leeds, UK), Cat. No. LM15
- LM24, Rat monoclonal, PlantProbes (Leeds, UK), Cat. No. LM24
- CCRC-M1, Mouse monoclonal, CarboSource (Athens, USA), Cat. No. CCRC-M1
- CCRC-M39, Mouse monoclonal, CarboSource (Athens, USA), Cat. No. CCRC-M39
- JIM4, Rat monoclonal, PlantProbes (Leeds, UK), Cat. No. JIM4
- JIM8, Rat monoclonal, PlantProbes (Leeds, UK), Cat. No. JIM8
- JIM14, Rat monoclonal, PlantProbes (Leeds, UK), Cat. No. JIM14
- JIM16, Rat monoclonal, PlantProbes (Leeds, UK), Cat. No. JIM16
- LM3, Rat monoclonal, PlantProbes (Leeds, UK), Cat. No. LM3
- JIM20, Rat monoclonal, PlantProbes (Leeds, UK), Cat. No. JIM20
- INRA-COU1, Mouse monoclonal, Institut National de la Recherche Agronomique (Nantes, France).
- LM11, Rat monoclonal, PlantProbes (Leeds, UK), Cat. No. LM11
- INRA-AX1, Mouse monoclonal, Institut National de la Recherche Agronomique (Nantes, France).
- Anti-rat antibody conjugated to alkaline phosphatase (here used as secondary antibody), Goat polyclonal, Sigma-Aldrich, Cat. No. A8438
- Anti-mouse antibody conjugated to alkaline phosphatase (here used as secondary antibody), Goat polyclonal, Sigma-Aldrich, Cat. No. A3562
- Anti-His tag antibody conjugated to alkaline phosphatase (here used as secondary antibody), Mouse monoclonal, Sigma-Aldrich, Cat. No. A5588
- Anti-rat antibody conjugated to FITC (here used as secondary antibody), Rabbit polyclonal, Sigma-Aldrich, Cat. No. F1763
- Anti-rat antibody conjugated to peroxidase (here used as secondary antibody), Goat polyclonal, Sigma-Aldrich, Cat. No. A9037

Validation

Validation of all primary monoclonal antibodies has been already performed. References of publications that validate their specificities are provided in Supplementary Table 1. All secondary antibodies stated above are commercially available and have been used in a large number of publications, thus they do not require additional validation.
