# Supplementary material for: Diatom fucan polysaccharide precipitates carbon during algal blooms
Source: Nat Commun. 2021 Feb 19;12:1150. doi: 10.1038/s41467-021-21009-6 (PMC7896085; doi:10.1038/s41467-021-21009-6)
Supplement: Supplementary file 5 — Description of Additional Supplementary Files [file 41467_2021_21009_MOESM5_ESM.pdf]

Description of Additional Supplementary File

Supplementary Data 1. Detected CAZymes and their specificities.

Table displays the carbohydrate-active enzymes (CAZymes) with relevance for carbohydrate degradation detected by our proteomic analysis. The annotation column indicates first the annotation of the gene according to Prokka, followed by best annotations from HMMER versus the dbCAN database and diamond BLAST against the CAZy database. Expression levels by date are shown as normalised spectral abundance factors x 100. CAZyme activities for each family are those listed on the CAZy website, http://www.cazy.org.
